# Supplementary material for: Reformed islets: a long-term primary cell platform for exploring mouse and human islet biology
Source: Cell Death Discov. 2024 Nov 23;10:480. doi: 10.1038/s41420-024-02234-6 (PMC11585622; doi:10.1038/s41420-024-02234-6)
Supplement: Supplementary file 1 — Supplementary Information [file 41420_2024_2234_MOESM1_ESM.pdf]

A

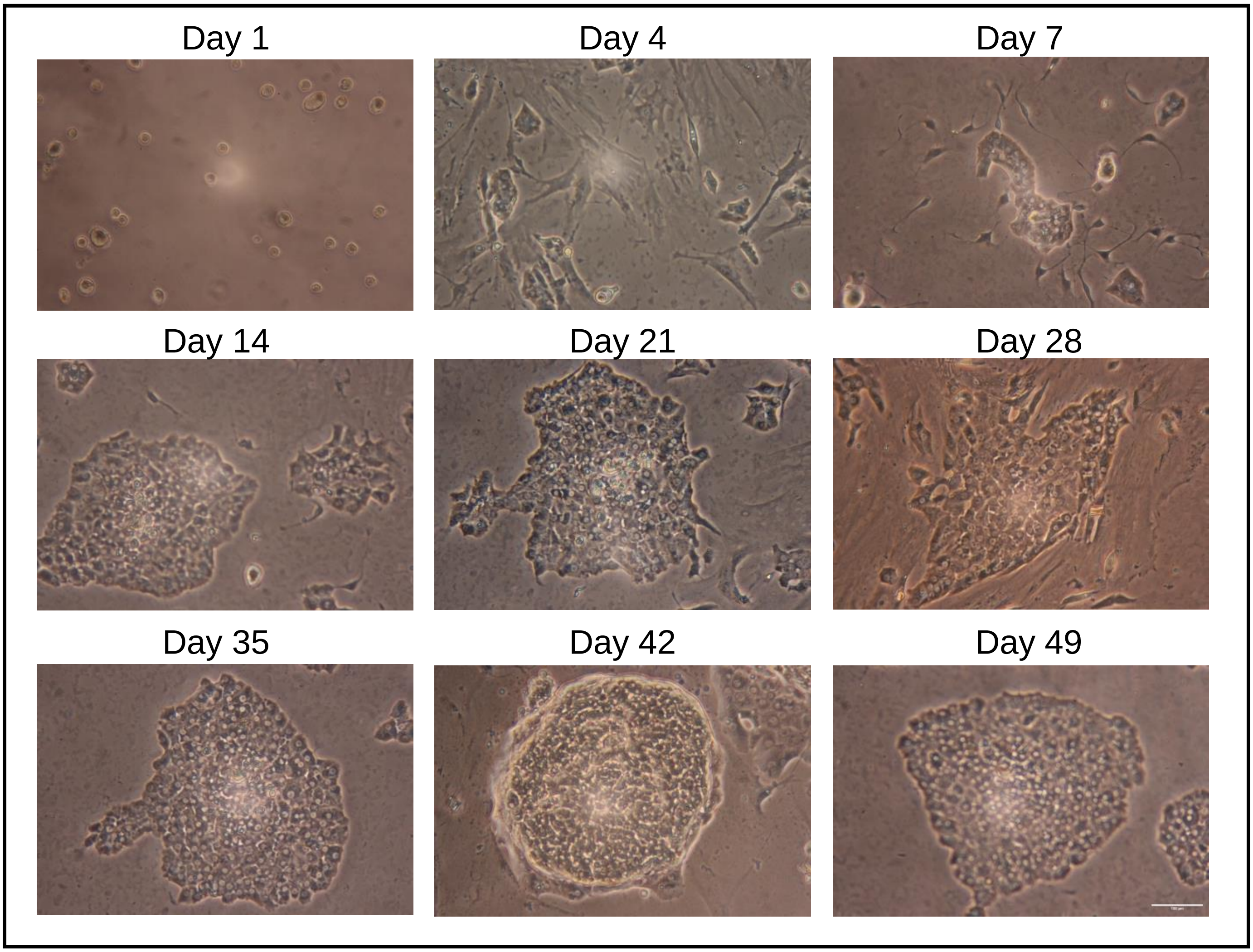

Fig. S1

A

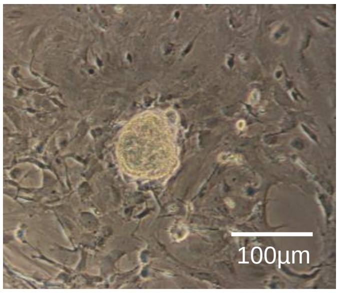

B

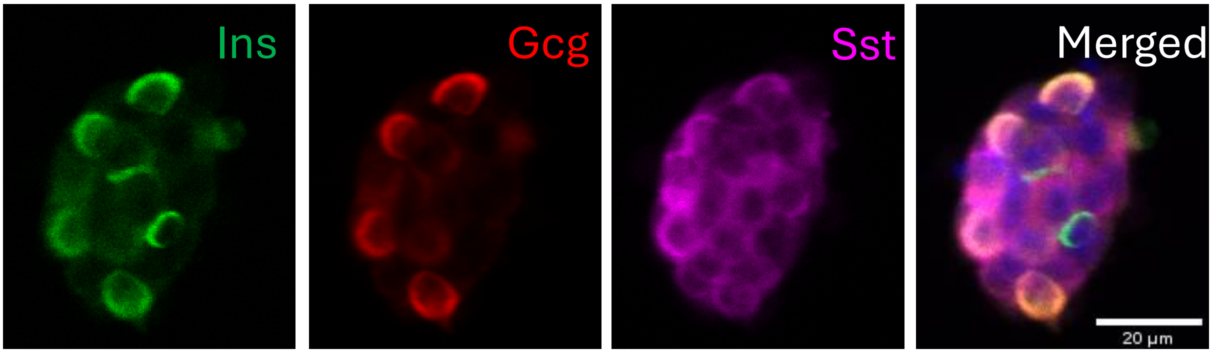

Fig. S2

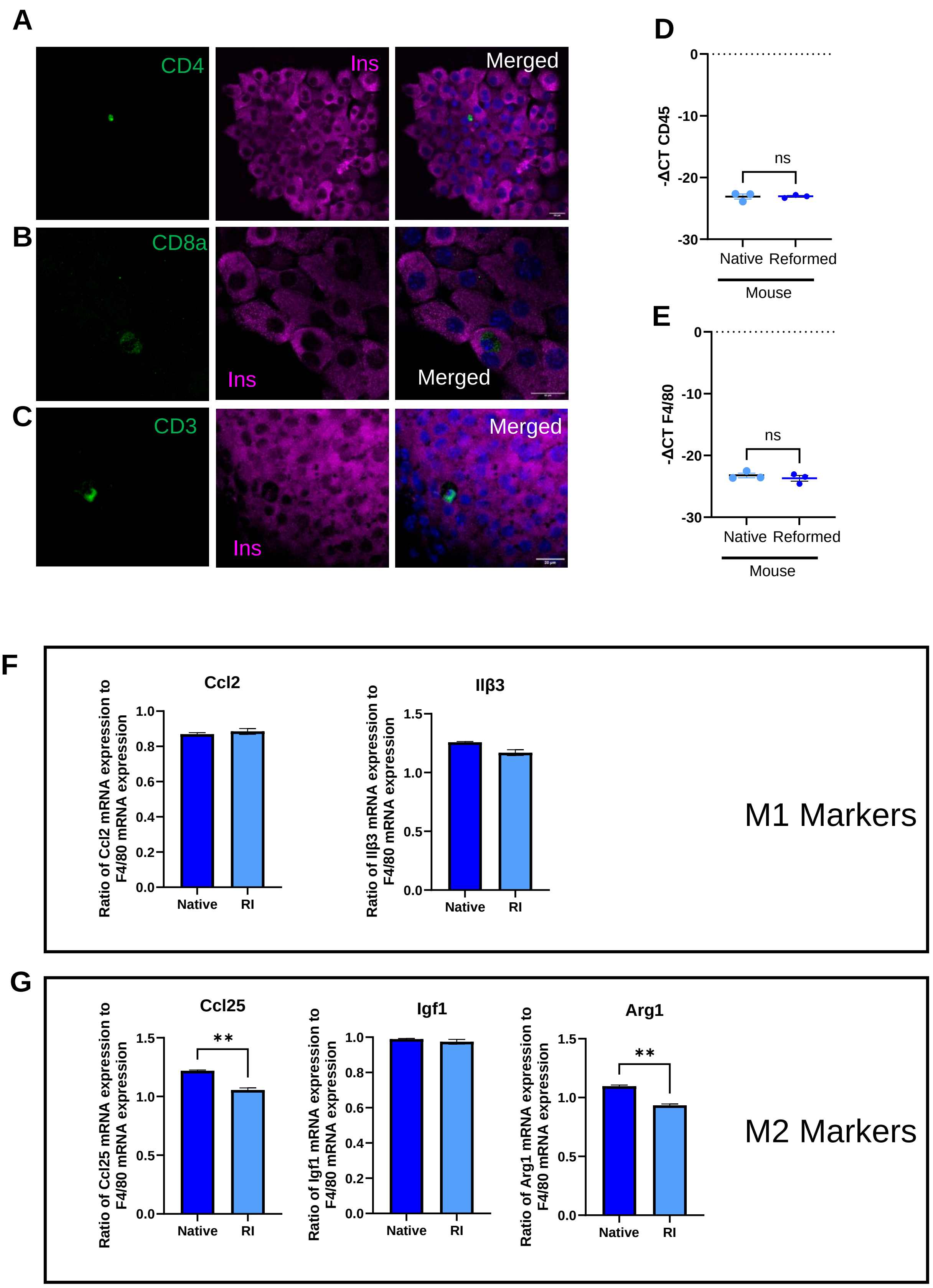
A
CD4
Ins
Merged
B
CD8a
Ins
Merged
C
CD3
Ins
Merged
D
-ΔCT CD45
0
-10
-20
-30
ns
Native
Reformed
Mouse
E
-ΔCT F4/80
0
-10
-20
-30
ns
Native
Reformed
Mouse
F
Ccl2
Ratio of Ccl2 mRNA expression to F4/80 mRNA expression
1.0
0.8
0.6
0.4
0.2
0.0
Native
RI
Ilβ3
Ratio of Ilβ3 mRNA expression to F4/80 mRNA expression
1.5
1.0
0.5
0.0
Native
RI
M1 Markers
G
Ccl25
Ratio of Ccl25 mRNA expression to F4/80 mRNA expression
1.5
1.0
0.5
0.0
**
Native
RI
Igf1
Ratio of Igf1 mRNA expression to F4/80 mRNA expression
1.0
0.8
0.6
0.4
0.2
0.0
Native
RI
Arg1
Ratio of Arg1 mRNA expression to F4/80 mRNA expression
1.5
1.0
0.5
0.0
**
Native
RI
M2 Markers

Fig. S3

A

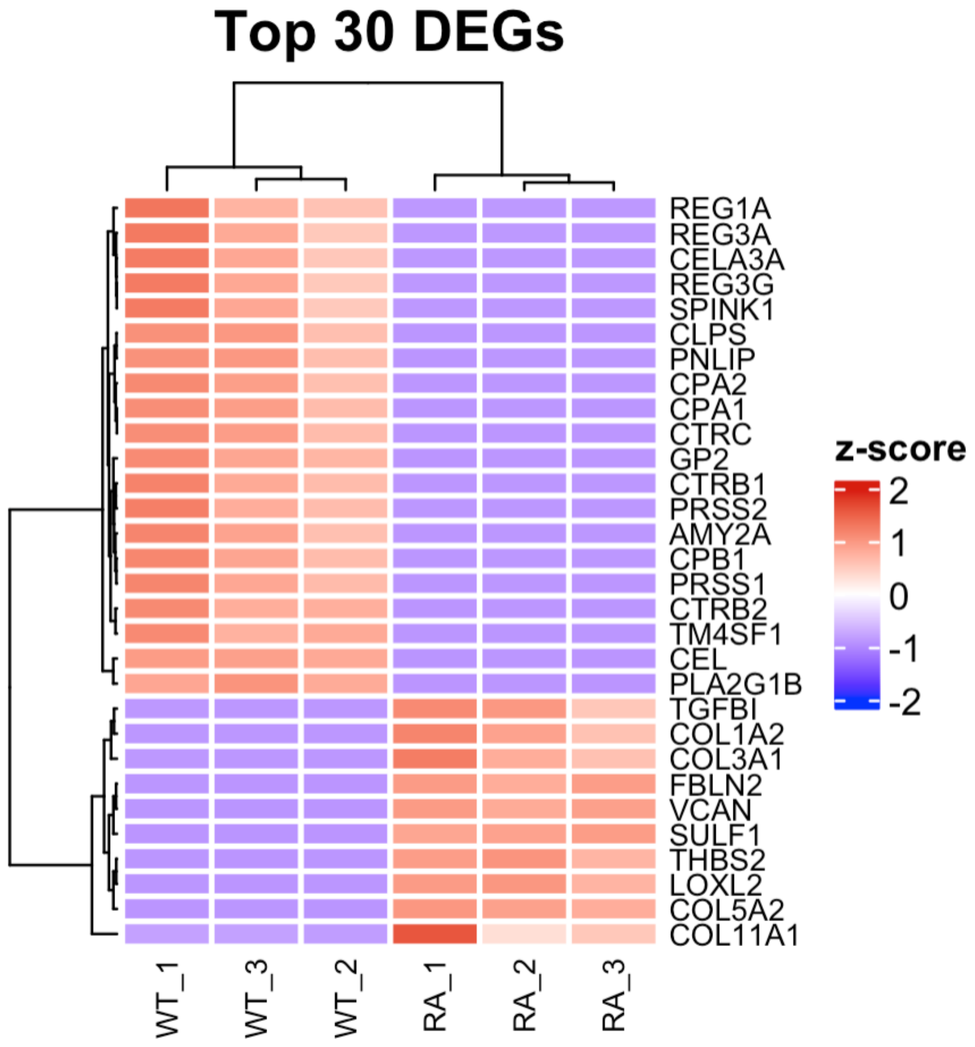
Top 30 DEGs
z-score
2
1
0
-1
-2
REG1A
REG3A
CELA3A
REG3G
SPINK1
CLPS
PNLIP
CPA2
CPA1
CTRC
GP2
CTRB1
PRSS2
AMY2A
CPB1
PRSS1
CTRB2
TM4SF1
CEL
PLA2G1B
TGFBI
COL1A2
COL3A1
FBLN2
VCAN
SULF1
THBS2
LOXL2
COL5A2
COL11A1
WT_1
WT_3
WT_2
RA_1
RA_2
RA_3

Fig. S4

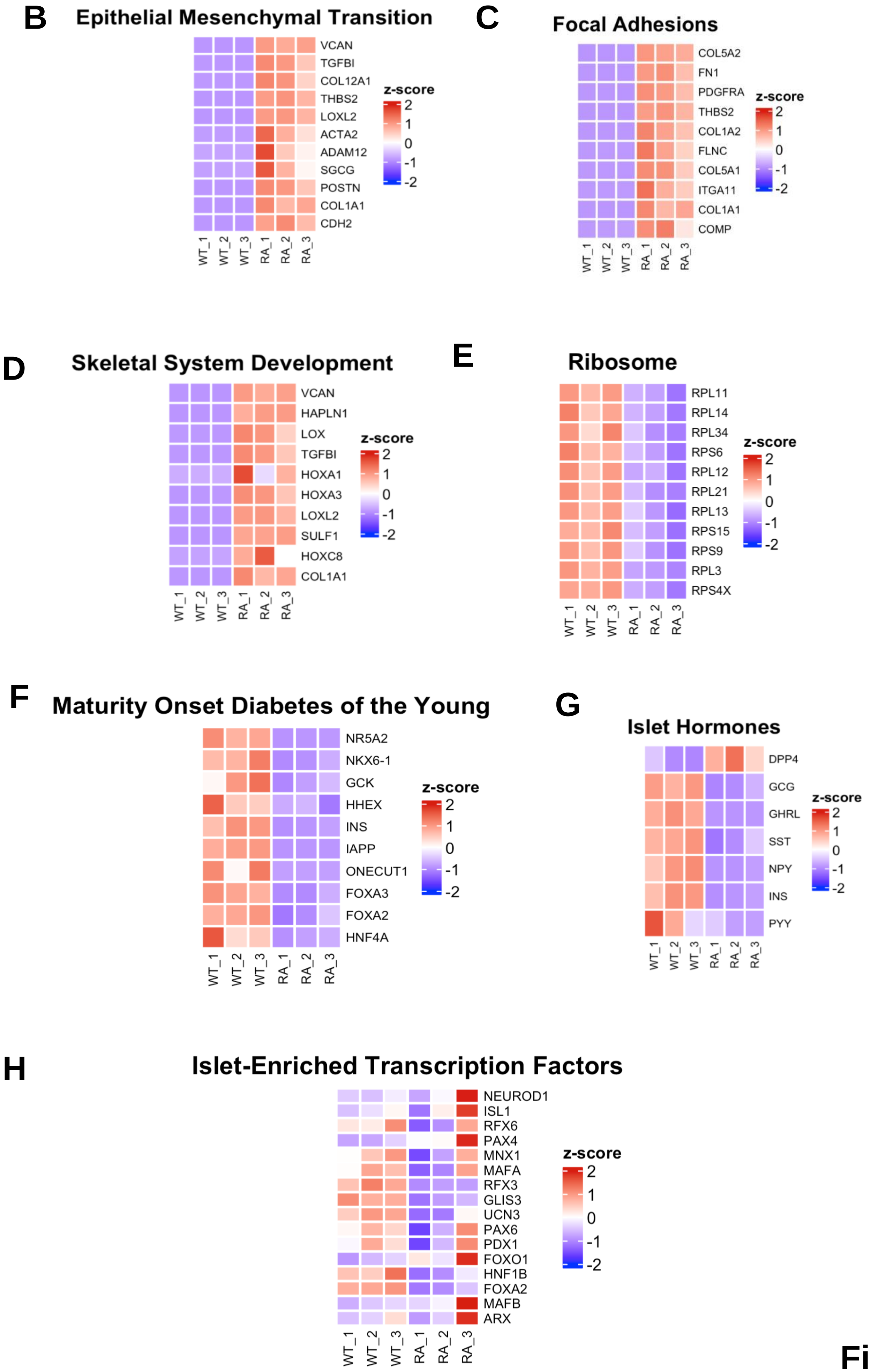
B
Epithelial Mesenchymal Transition
VCAN
TGFBI
COL12A1
THBS2
LOXL2
ACTA2
ADAM12
SGCG
POSTN
COL1A1
CDH2
z-score
2
1
0
-1
-2
WT_1
WT_2
WT_3
RA_1
RA_2
RA_3
C
Focal Adhesions
COL5A2
FN1
PDGFRA
THBS2
COL1A2
FLNC
COL5A1
ITGA11
COL1A1
COMP
z-score
2
1
0
-1
-2
WT_1
WT_2
WT_3
RA_1
RA_2
RA_3
D
Skeletal System Development
VCAN
HAPLN1
LOX
TGFBI
HOXA1
HOXA3
LOXL2
SULF1
HOXC8
COL1A1
z-score
2
1
0
-1
-2
WT_1
WT_2
WT_3
RA_1
RA_2
RA_3
E
Ribosome
RPL11
RPL14
RPL34
RPS6
RPL12
RPL21
RPL13
RPS15
RPS9
RPL3
RPS4X
z-score
2
1
0
-1
-2
WT_1
WT_2
WT_3
RA_1
RA_2
RA_3
F
Maturity Onset Diabetes of the Young
NR5A2
NKX6-1
GCK
HHEX
INS
IAPP
ONECUT1
FOXA3
FOXA2
HNF4A
z-score
2
1
0
-1
-2
WT_1
WT_2
WT_3
RA_1
RA_2
RA_3
G
Islet Hormones
DPP4
GCG
GHRL
SST
NPY
INS
PYY
z-score
2
1
0
-1
-2
WT_1
WT_2
WT_3
RA_1
RA_2
RA_3
H
Islet-Enriched Transcription Factors
NEUROD1
ISL1
RFX6
PAX4
MNX1
MAFA
RFX3
GLIS3
UCN3
PAX6
PDX1
FOXO1
HNF1B
FOXA2
MAFB
ARX
z-score
2
1
0
-1
-2
WT_1
WT_2
WT_3
RA_1
RA_2
RA_3

Fig. S4

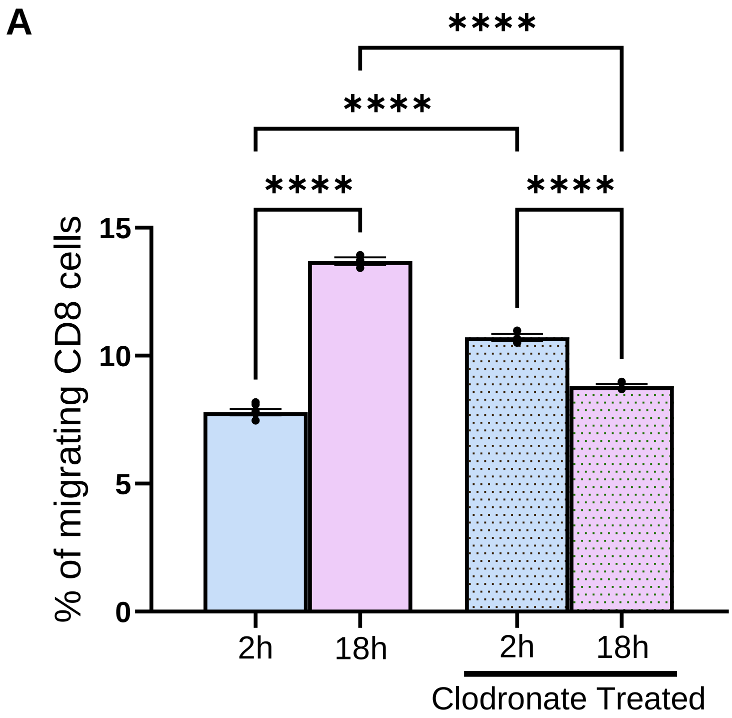
A
****
****
****
****
% of migrating CD8 cells
15
10
5
0
2h
18h
2h
18h
Clodronate Treated

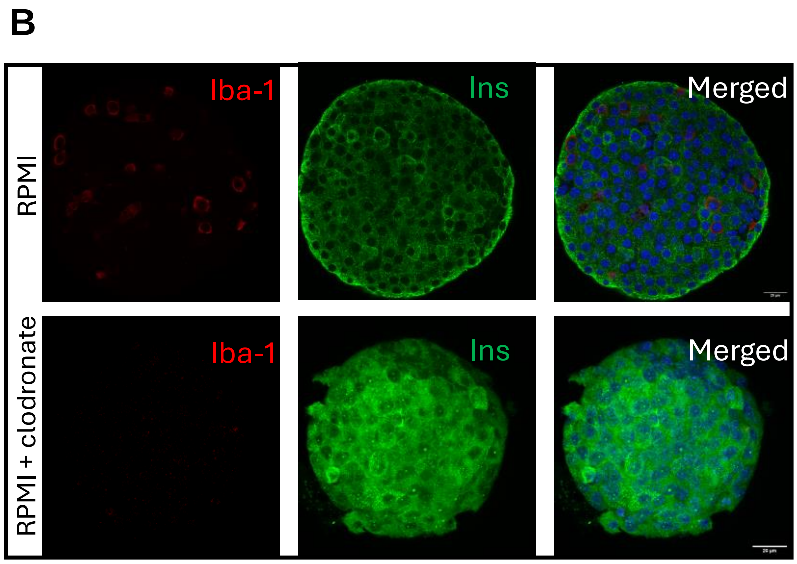
B
RPMI
RPMI + clodronate
Iba-1
Ins
Merged
Iba-1
Ins
Merged

Fig. S5

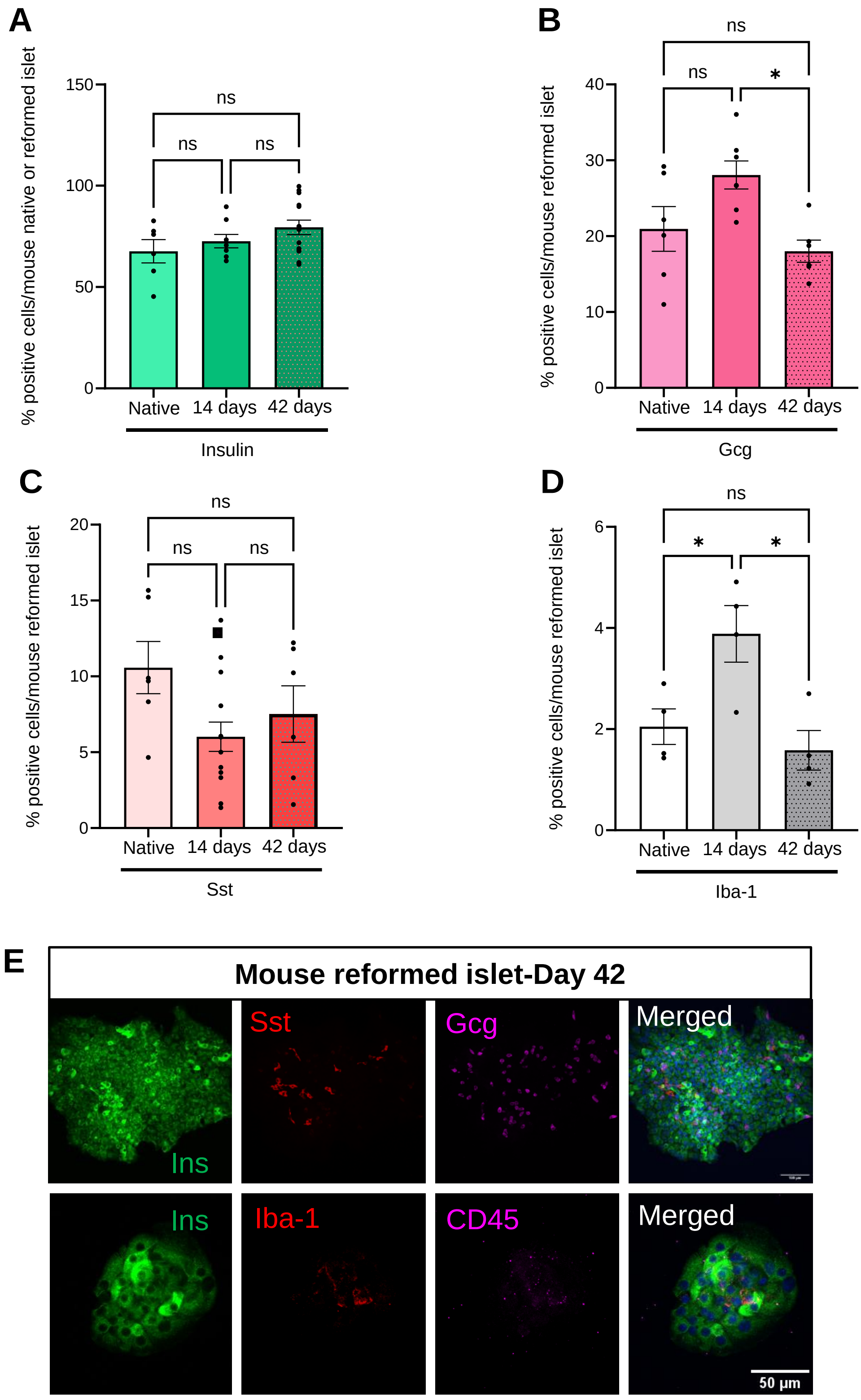
A
% positive cells/mouse native or reformed islet
150
100
50
0
ns
ns
ns
Native
14 days
42 days
Insulin
B
% positive cells/mouse reformed islet
40
30
20
10
0
ns
ns
*
Native
14 days
42 days
Gcg
C
% positive cells/mouse reformed islet
20
15
10
5
0
ns
ns
ns
Native
14 days
42 days
Sst
D
% positive cells/mouse reformed islet
6
4
2
0
ns
*
*
Native
14 days
42 days
Iba-1
E
Mouse reformed islet-Day 42
Ins
Sst
Gcg
Merged
Ins
Iba-1
CD45
Merged
50 µm

Fig. S6

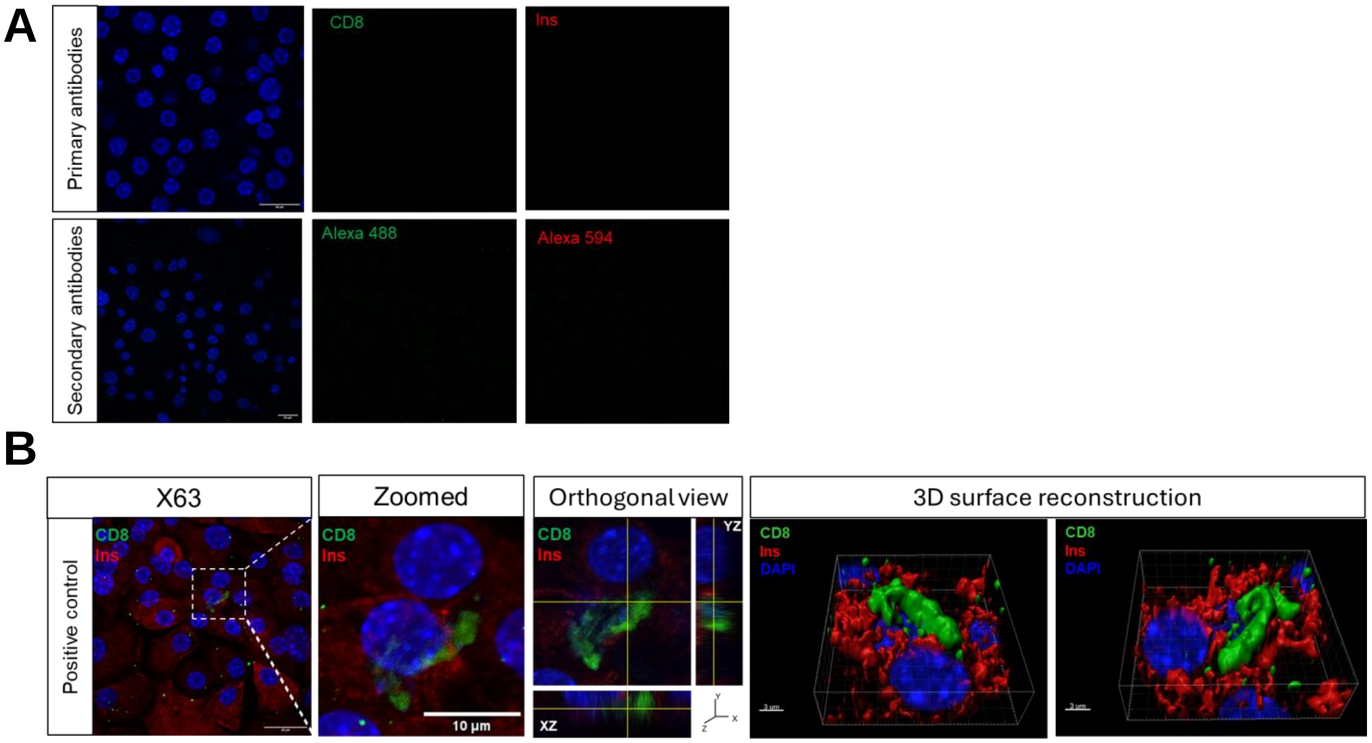
A
Primary antibodies
CD8
Ins
Secondary antibodies
Alexa 488
Alexa 594
B
X63
Zoomed
Orthogonal view
3D surface reconstruction
Positive control
CD8
Ins
DAPI
10 µm
YZ
XZ

Fig. S7

Supplementary Figure Legends

Figure S1. Brightfield images of reformed islets from day 1 to day 49. Light microscopy images illustrate the formation reformed islets from day 1. By day 14 the islets are fully formed and are maintained till day 49.

Figure S2. Reformed islet formation after 7 days. Light microscopy images show that mouse islet cells formed 3-dimensional spheroid structures after 7 days of seeding. Scale bar is 100µm (A). Immunofluorescence detection by confocal microscopy of islet hormones in maturing mouse reformed at day 7 confirmed that developing reformed islets expressed insulin (Ins; green), glucagon (Gcg; red) and somatostatin (Sst; purple). Scale bar is 20µm.

Figure S3. Immune population in reformed islets. Immunostaining of the reformed islets with antibodies directed against insulin (Ins; purple) and immune markers CD4 (A), CD8a (B) and CD3 (C) (green) confirmed the presence of both resident macrophages and resident T-cells in reformed islets. Scale bar is 20µm. (D, E) Negative delta Ct values distribution graphs of Ptprc (CD45, all immune cell marker) and Adgre1(F4/80, macrophage marker) in native and reformed mouse islets detected by quantitative PCR. The macrophage polarity (pro-inflammatory-M1, anti-inflammatory-M2) of reformed islets are evaluated by quantitative PCR of *Ccl2*, and *Ilβ3* (M1) (F) and *Ccl25*, *Igf1* and *Arg1* (M2) (G). Quantification of M1 and M2 markers are mean ± SEM relative to F4/80 mRNA expression of native and reformed islets respectively. $^{**}p < 0.01$; unpaired t test with Welch's correction.

Figure S4. Transcriptomic analyses of native and reformed human islets. (A) Heatmap illustrating top 30 differentially expressed genes in reformed islets. (B, C, D) Heatmaps illustrating genes differentially enriched in EMT, Focal adhesion and Skeletal development pathway process. (E) KEGG enriched pathways related to ribosome biogenesis. (F) Pathways enriched in Maturity Onset of the Diabetes of the Young. (G) Heatmap of mRNA expression of islets hormones. (H) Heatmap of selected subset of islet-enriched transcription factors between native and reformed islets.

Figure S5. Depletion of resident macrophages affect migratory response of T cells. Mouse reformed islets derived from BALB/c were exposed to 600ug/ml of clodronate liposome for 48h before co-culturing them with haplotype matched $CD8^+$T cells, derived from diabetic NOD mice. Bar graph shows mean total percentage of $CD8^+$T cells migrating when cocultured with haplotype matched BALB/c reformed islets (S A). Two-way ANOVA or unpaired Student t-test were performed to assess significance. $^{****}p < 0.0001$. (B) represents images of invasion of reformed islets in presence and absence of $CD8^+$T cells. In invasion experiments, the reformed islets were immunolabelled with antibodies against insulin (Ins; purple), CD8

(green), CD80 (red) and a nuclear stain DAPI. Scale bars represent 20µm for fluorescence microscopy.

Figure S6. Comparative and quantitative analyses of cell composition of native islets, reformed islets at day14 and day 42.

Percentages of different cell types in mouse reformed islets at day 14 and day 42 were calculated using the cell counter plugin in ImageJ software and compared to native mouse islets (A-D). Numerical data are presented as the mean ± SEM, n=4-14 observations (no. of islets per experiment) in 6-10 coverslips containing reformed islets. *ns* represents p>0.05, unpaired Student t-test or One-way ANOVA, Šídák's multiple comparisons test. Reformed islets at day 42 were immunolabelled for insulin (Ins; green), somatostatin (Sst; red), glucagon (Gcg; purple) (E-upper panel), and resident macrophages (Iba-1; red) (E-lower panel). DAPI nuclear staining is shown in blue. Scale bars are 100 µm (upper panel) and 50 µm (lower panel).

Figure S7. The primary, secondary and positive controls to determine the specificities of antibodies.

The primary antibodies alone, secondary antibodies and positive control indicate the specificity of the primary antibodies. For positive control, reformed islets were immunolabelled with CD8, insulin and counterstained with DAPI. (A) The first panel illustrates reformed islets that were probed with primary antibodies while the second panel shows reformed islets treated with secondary antibodies only (Alexa 488 and Alexa 594). (B) For positive control, reformed islets were immunolabelled with CD8, insulin and counterstained with DAPI. The orthogonal views represent the XZ and the YZ planes of the positive control (B) while the 3D surface reconstruction illustrates the position of CD8 cells and insulin positive cells (C).
